# (2*E*)-1-(4-Bromo­phen­yl)-3-[3-(4-meth­oxy­phen­yl)-1-phenyl-1*H*-pyrazol-4-yl]prop-2-en-1-one

**DOI:** 10.1107/S1600536813016838

**Published:** 2013-06-22

**Authors:** R. Prasath, P. Bhavana, Seik Weng Ng, Edward R. T. Tiekink

**Affiliations:** a Department of Chemistry, BITS, Pilani - K. K. Birla Goa Campus, Goa 403 726, India; b Department of Chemistry, University of Malaya, 50603 Kuala Lumpur, Malaysia; c Chemistry Department, Faculty of Science, King Abdulaziz University, PO Box 80203 Jeddah, Saudi Arabia

## Abstract

The pyrazole ring in the title compound, C_25_H_19_BrN_2_O_2_, is almost planar (r.m.s. deviation = 0.003 Å) and forms dihedral angles of 7.56 (13) and 56.48 (13)° with the N- and C-bound benzene rings, respectively. The prop-2-en-1-one residue has an *E* conformation about the C=C double bond [1.328 (4) Å] and is almost coplanar with the pyrazole ring [C—C—C—C torsion angle = −174.4 (3)°]. A twist between the prop-2-en-1-one unit and the terminal benzene ring is evident [C—C—C—C torsion angle = −15.4 (4)°]. In the crystal, mol­ecules are consolidated into a three-dimensional architecture by C—H⋯O, C—H⋯π and π–π [centroid–centroid separation = 3.7597 (16) Å] inter­actions.

## Related literature

For background details and biological applications of pyrazole and chalcones, see: Babasaheb *et al.* (2009 ▶); Prasath & Bhavana (2012 ▶); Prasath *et al.* (2013 ▶). For the structure of the 4-meth­oxy­phenyl pyrazole compound, see: Fun *et al.* (2011 ▶).
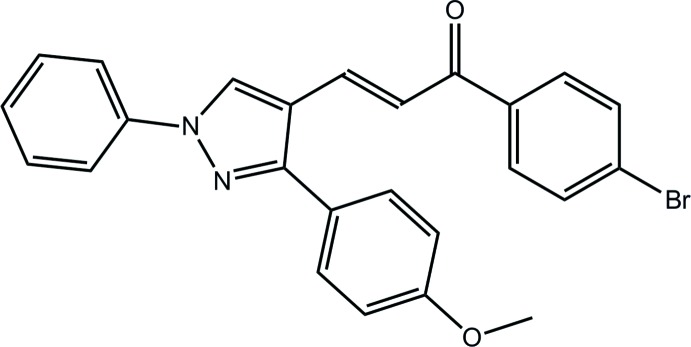


## Experimental

### 

#### Crystal data

C_25_H_19_BrN_2_O_2_
*M* *_r_* = 459.33
Triclinic, 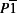

*a* = 7.3643 (3) Å
*b* = 10.6795 (5) Å
*c* = 13.1038 (6) Å
α = 91.822 (4)°
β = 101.311 (4)°
γ = 91.792 (3)°
*V* = 1009.31 (8) Å^3^
*Z* = 2
Mo *K*α radiation
μ = 2.06 mm^−1^
*T* = 100 K
0.50 × 0.40 × 0.30 mm

#### Data collection

Agilent SuperNova Dual diffractometer with an Atlas detector
Absorption correction: multi-scan (*CrysAlis PRO*; Agilent, 2013 ▶) *T*_min_ = 0.921, *T*_max_ = 1.000
8736 measured reflections
4649 independent reflections
3875 reflections with *I* > 2σ(*I*)
*R*_int_ = 0.038

#### Refinement

*R*[*F* ^2^ > 2σ(*F* ^2^)] = 0.043
*wR*(*F* ^2^) = 0.111
*S* = 1.04
4649 reflections
271 parameters
H-atom parameters constrained
Δρ_max_ = 0.47 e Å^−3^
Δρ_min_ = −0.55 e Å^−3^

### 

Data collection: *CrysAlis PRO* (Agilent, 2013 ▶); cell refinement: *CrysAlis PRO*; data reduction: *CrysAlis PRO*; program(s) used to solve structure: *SHELXS97* (Sheldrick, 2008 ▶); program(s) used to refine structure: *SHELXL97* (Sheldrick, 2008 ▶); molecular graphics: *ORTEP-3 for Windows* (Farrugia, 2012 ▶) and *DIAMOND* (Brandenburg, 2006 ▶); software used to prepare material for publication: *publCIF* (Westrip, 2010 ▶).

## Supplementary Material

Crystal structure: contains datablock(s) global, I. DOI: 10.1107/S1600536813016838/hb7095sup1.cif

Structure factors: contains datablock(s) I. DOI: 10.1107/S1600536813016838/hb7095Isup2.hkl

Supplementary material file. DOI: 10.1107/S1600536813016838/hb7095Isup3.cml

Additional supplementary materials:  crystallographic information; 3D view; checkCIF report

## Figures and Tables

**Table 1:** Hydrogen-bond geometry (Å, °) *Cg*1 is the centroid of the C1–C6 ring.

*D*—H⋯*A*	*D*—H	H⋯*A*	*D*⋯*A*	*D*—H⋯*A*
C11—H11⋯O1^i^	0.95	2.25	3.198 (3)	173
C25—H25*B*⋯*Cg*1^ii^	0.98	2.61	3.478 (3)	148

Symmetry codes: (i) 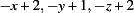; (ii) 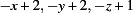.

**Fig. 1.**
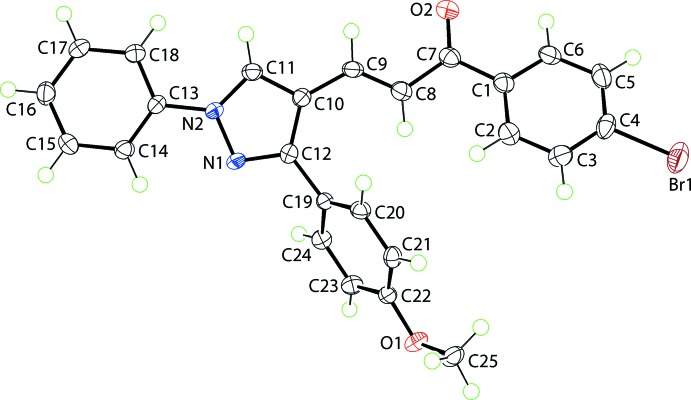
The molecular structure of (I) showing displacement ellipsoids at the 70% probability level.

**Fig. 2.**
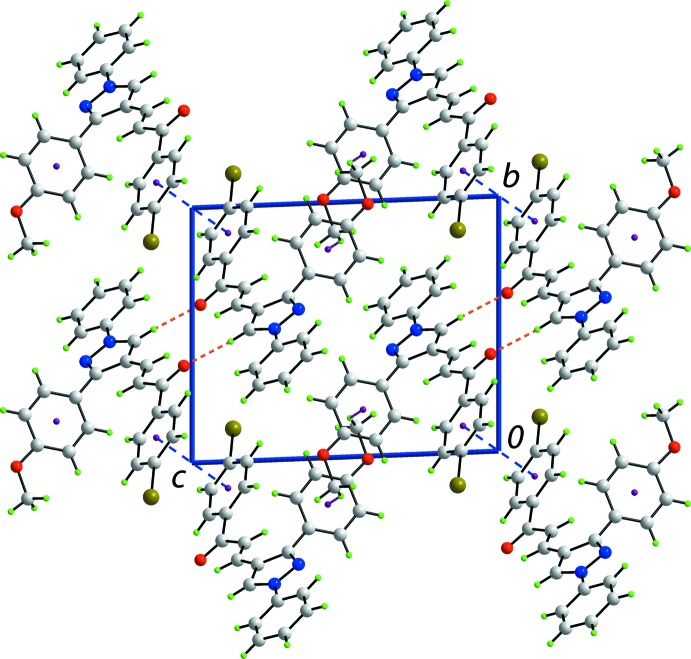
A view in projection down the a axis of the unit-cell contents of (I). The C—H···O, C—H···π, and π—π interactions are shown as blue, purple and blue dashed lines, respectively.

## supplementary crystallographic information

### Comment

In addition to their being valuable intermediates in organic synthesis (Prasath & Bhavana, 2012), nitrogen-containing heterocyclic analogues, such as pyrazole chalcones, exhibit a variety of biological activities, *e.g*. anti-plasmodial, anti-microbial and anti-cancer activities (Prasath *et al.*, 2013; Babasaheb *et al.*, 2009). It was in this context that the title compound, (I), was investigated.

The molecular structure of (I), Fig. 1, comprises a planar (r.m.s. deviation = 0.003 Å) tri-substituted pyrazoyl ring. The N1- and C12-bound benzene rings form dihedral angles of 7.56 (13) and 56.48 (13)°, respectively, with the five-membered ring, and 60.88 (12)° with each other. The prop-2-en-1-one residue has an *E*-configuration about the C8═C9 double bond [1.328 (4) Å], and is also co-planar with the five-membered ring as seen in the value of the C8—C9—C10—C11 torsion angle of -174.4 (3)°. This planarity does not extend to the terminal benzene ring as there is a twist manifested in the C2—C1—C7—C8 torsion angle of -15.4 (4)°. The observed conformation for (I) resembles closely that reported recently for the unsubstituted parent compound (Fun *et al.*, 2011).

In the crystal packing, centrosymmetrically related molecules are connected *via* C—H···O interactions, Table 1, and these are connected into a three-dimensional architecture by π—π [between centrosymmetrically related bromobenzene rings; 3.7597 (16)° for symmetry operation 1 - *x*, 2 - *y*, 2 - *z*] and methyl-C—H···π(methoxybenzene ring) interactions, Fig. 2 and Table 1.

### Experimental

A mixture of 3-(4-methoxyphenyl)-1-phenyl-*1H*-pyrazole-4-carbaldehyde (1.4 g, 0.005 *M*), 4-bromo acetophenone (1 g, 0.005 *M*) and KOH (0.5 g) in distilled ethanol (50 ml) was stirred for 12 h at room temperature. The resulting mixture was neutralized with dilute acetic acid. The resultant solid was filtered, dried and purified by column chromatography using 1:2 mixture of ethyl acetate and hexane. Re-crystallization was by slow evaporation of an acetone solution of (I) which yielded yellow needles. *M*.pt. 433–435 K. Yield: 80%.

### Refinement

The C-bound H atoms were geometrically placed (C—H = 0.95–0.98 Å) and refined as riding with *U_iso_*(H) = 1.2–1.5*U_eq_*(C).

### Crystal data

**Table:**

C_25_H_19_BrN_2_O_2_	*Z* = 2
*M**_r_* = 459.33	*F*(000) = 468
Triclinic, *P*1	*D*_x_ = 1.511 Mg m^−^^3^
Hall symbol: -P 1	Mo *K*α radiation, λ = 0.71073 Å
*a* = 7.3643 (3) Å	Cell parameters from 2849 reflections
*b* = 10.6795 (5) Å	θ = 3.0–27.5°
*c* = 13.1038 (6) Å	µ = 2.06 mm^−^^1^
α = 91.822 (4)°	*T* = 100 K
β = 101.311 (4)°	Prism, yellow
γ = 91.792 (3)°	0.50 × 0.40 × 0.30 mm
*V* = 1009.31 (8) Å^3^	

### Data collection

**Table:**

Agilent SuperNova Dual diffractometer with an Atlas detector	4649 independent reflections
Radiation source: SuperNova (Mo) X-ray Source	3875 reflections with *I* > 2σ(*I*)
Mirror monochromator	*R*_int_ = 0.038
Detector resolution: 10.4041 pixels mm^-1^	θ_max_ = 27.6°, θ_min_ = 3.0°
ω scan	*h* = −9→9
Absorption correction: multi-scan (*CrysAlis PRO*; Agilent, 2013)	*k* = −13→13
*T*_min_ = 0.921, *T*_max_ = 1.000	*l* = −15→17
8736 measured reflections	

### Refinement

**Table:**

Refinement on *F*^2^	Primary atom site location: structure-invariant direct methods
Least-squares matrix: full	Secondary atom site location: difference Fourier map
*R*[*F*^2^ > 2σ(*F*^2^)] = 0.043	Hydrogen site location: inferred from neighbouring sites
*wR*(*F*^2^) = 0.111	H-atom parameters constrained
*S* = 1.04	*w* = 1/[σ^2^(*F*_o_^2^) + (0.0485*P*)^2^ + 0.731*P*] where *P* = (*F*_o_^2^ + 2*F*_c_^2^)/3
4649 reflections	(Δ/σ)_max_ = 0.001
271 parameters	Δρ_max_ = 0.47 e Å^−^^3^
0 restraints	Δρ_min_ = −0.55 e Å^−^^3^

### Special details

**Table:**

Geometry. All e.s.d.'s (except the e.s.d. in the dihedral angle between two l.s. planes) are estimated using the full covariance matrix. The cell e.s.d.'s are taken into account individually in the estimation of e.s.d.'s in distances, angles and torsion angles; correlations between e.s.d.'s in cell parameters are only used when they are defined by crystal symmetry. An approximate (isotropic) treatment of cell e.s.d.'s is used for estimating e.s.d.'s involving l.s. planes.
Refinement. Refinement of *F*^2^ against ALL reflections. The weighted *R*-factor *wR* and goodness of fit *S* are based on *F*^2^, conventional *R*-factors *R* are based on *F*, with *F* set to zero for negative *F*^2^. The threshold expression of *F*^2^ > σ(*F*^2^) is used only for calculating *R*-factors(gt) *etc*. and is not relevant to the choice of reflections for refinement. *R*-factors based on *F*^2^ are statistically about twice as large as those based on *F*, and *R*- factors based on ALL data will be even larger.

### Fractional atomic coordinates and isotropic or equivalent isotropic displacement parameters (Å^2^)

**Table:**

	*x*	*y*	*z*	*U*_iso_*/*U*_eq_	
Br1	0.11696 (4)	1.12761 (3)	0.86611 (2)	0.03080 (12)	
O1	0.6500 (3)	0.61824 (19)	0.97288 (15)	0.0248 (4)	
O2	0.6830 (2)	0.98810 (16)	0.42732 (14)	0.0185 (4)	
N1	1.2201 (3)	0.58644 (19)	0.65301 (17)	0.0145 (4)	
N2	1.3006 (3)	0.51261 (19)	0.73128 (16)	0.0137 (4)	
C1	0.5191 (4)	0.7966 (2)	0.8896 (2)	0.0172 (5)	
C2	0.5417 (4)	0.8979 (3)	0.8283 (2)	0.0213 (6)	
H2	0.6396	0.8992	0.7906	0.026*	
C3	0.4235 (4)	0.9967 (3)	0.8216 (2)	0.0227 (6)	
H3	0.4391	1.0656	0.7796	0.027*	
C4	0.2828 (4)	0.9933 (3)	0.8769 (2)	0.0208 (6)	
C5	0.2543 (4)	0.8941 (3)	0.9378 (2)	0.0227 (6)	
H5	0.1551	0.8931	0.9745	0.027*	
C6	0.3751 (4)	0.7954 (3)	0.9440 (2)	0.0205 (6)	
H6	0.3587	0.7266	0.9859	0.025*	
C7	0.6504 (4)	0.6906 (2)	0.9023 (2)	0.0173 (5)	
C8	0.7763 (4)	0.6782 (2)	0.8287 (2)	0.0176 (5)	
H8	0.7483	0.7174	0.7637	0.021*	
C9	0.9287 (4)	0.6129 (2)	0.8514 (2)	0.0164 (5)	
H9	0.9515	0.5759	0.9174	0.020*	
C10	1.0640 (3)	0.5920 (2)	0.7864 (2)	0.0147 (5)	
C11	1.2115 (4)	0.5143 (2)	0.8117 (2)	0.0155 (5)	
H11	1.2439	0.4703	0.8742	0.019*	
C12	1.0770 (3)	0.6352 (2)	0.68607 (19)	0.0130 (5)	
C13	1.4631 (3)	0.4466 (2)	0.7208 (2)	0.0142 (5)	
C14	1.5469 (4)	0.4699 (2)	0.6376 (2)	0.0162 (5)	
H14	1.4993	0.5305	0.5889	0.019*	
C15	1.7022 (4)	0.4038 (2)	0.6258 (2)	0.0172 (5)	
H15	1.7594	0.4183	0.5681	0.021*	
C16	1.7731 (4)	0.3171 (2)	0.6979 (2)	0.0198 (6)	
H16	1.8791	0.2722	0.6899	0.024*	
C17	1.6886 (4)	0.2960 (3)	0.7821 (2)	0.0215 (6)	
H17	1.7381	0.2370	0.8319	0.026*	
C18	1.5322 (4)	0.3604 (2)	0.7942 (2)	0.0186 (5)	
H18	1.4741	0.3457	0.8516	0.022*	
C19	0.9641 (3)	0.7257 (2)	0.62029 (19)	0.0132 (5)	
C20	0.9485 (3)	0.8468 (2)	0.6597 (2)	0.0153 (5)	
H20	1.0031	0.8678	0.7303	0.018*	
C21	0.8557 (3)	0.9374 (2)	0.5987 (2)	0.0153 (5)	
H21	0.8458	1.0191	0.6274	0.018*	
C22	0.7771 (3)	0.9074 (2)	0.4951 (2)	0.0141 (5)	
C23	0.7907 (4)	0.7859 (2)	0.4545 (2)	0.0164 (5)	
H23	0.7359	0.7648	0.3840	0.020*	
C24	0.8836 (3)	0.6967 (2)	0.5167 (2)	0.0154 (5)	
H24	0.8925	0.6146	0.4884	0.019*	
C25	0.6747 (4)	1.1152 (2)	0.4644 (2)	0.0196 (6)	
H25A	0.6039	1.1636	0.4089	0.029*	
H25B	0.8006	1.1521	0.4851	0.029*	
H25C	0.6138	1.1166	0.5245	0.029*	

### Atomic displacement parameters (Å^2^)

**Table:**

	*U*^11^	*U*^22^	*U*^33^	*U*^12^	*U*^13^	*U*^23^
Br1	0.02570 (17)	0.03300 (19)	0.03209 (19)	0.01428 (13)	0.00041 (12)	−0.00323 (13)
O1	0.0285 (11)	0.0290 (11)	0.0198 (10)	0.0076 (9)	0.0099 (9)	0.0081 (9)
O2	0.0181 (9)	0.0157 (9)	0.0203 (9)	0.0013 (7)	−0.0009 (8)	0.0053 (8)
N1	0.0168 (10)	0.0125 (10)	0.0143 (10)	0.0032 (8)	0.0026 (8)	0.0040 (8)
N2	0.0139 (10)	0.0133 (10)	0.0140 (10)	0.0025 (8)	0.0023 (8)	0.0037 (8)
C1	0.0174 (12)	0.0205 (13)	0.0129 (12)	0.0009 (11)	0.0018 (10)	−0.0020 (10)
C2	0.0200 (13)	0.0246 (14)	0.0206 (14)	0.0025 (11)	0.0065 (11)	0.0015 (12)
C3	0.0259 (14)	0.0216 (14)	0.0209 (14)	0.0038 (12)	0.0042 (12)	0.0041 (12)
C4	0.0177 (13)	0.0239 (14)	0.0185 (13)	0.0078 (11)	−0.0025 (11)	−0.0043 (11)
C5	0.0157 (13)	0.0336 (16)	0.0189 (14)	0.0042 (12)	0.0043 (11)	−0.0035 (12)
C6	0.0212 (13)	0.0268 (14)	0.0147 (13)	0.0023 (11)	0.0054 (11)	0.0038 (11)
C7	0.0172 (12)	0.0200 (13)	0.0144 (12)	−0.0010 (10)	0.0025 (10)	−0.0003 (11)
C8	0.0195 (13)	0.0189 (13)	0.0149 (12)	0.0006 (11)	0.0044 (10)	0.0007 (10)
C9	0.0192 (12)	0.0180 (12)	0.0124 (12)	0.0009 (10)	0.0034 (10)	0.0039 (10)
C10	0.0141 (12)	0.0149 (12)	0.0151 (12)	0.0014 (10)	0.0024 (10)	0.0015 (10)
C11	0.0170 (12)	0.0155 (12)	0.0144 (12)	0.0003 (10)	0.0032 (10)	0.0037 (10)
C12	0.0111 (11)	0.0113 (11)	0.0157 (12)	−0.0011 (9)	0.0013 (9)	−0.0006 (10)
C13	0.0139 (11)	0.0136 (11)	0.0144 (12)	0.0025 (10)	0.0009 (10)	0.0011 (10)
C14	0.0189 (12)	0.0140 (12)	0.0160 (12)	0.0025 (10)	0.0040 (10)	0.0016 (10)
C15	0.0174 (12)	0.0195 (13)	0.0158 (13)	−0.0001 (10)	0.0060 (10)	−0.0008 (10)
C16	0.0172 (12)	0.0191 (13)	0.0244 (14)	0.0043 (11)	0.0067 (11)	0.0013 (11)
C17	0.0231 (14)	0.0217 (14)	0.0210 (14)	0.0090 (11)	0.0053 (11)	0.0090 (11)
C18	0.0190 (13)	0.0186 (13)	0.0200 (13)	0.0036 (11)	0.0064 (11)	0.0073 (11)
C19	0.0109 (11)	0.0152 (12)	0.0142 (12)	0.0009 (9)	0.0034 (9)	0.0035 (10)
C20	0.0155 (12)	0.0185 (12)	0.0116 (12)	0.0004 (10)	0.0018 (10)	0.0013 (10)
C21	0.0145 (12)	0.0126 (12)	0.0190 (13)	0.0006 (10)	0.0046 (10)	−0.0011 (10)
C22	0.0088 (11)	0.0157 (12)	0.0185 (13)	−0.0015 (9)	0.0038 (10)	0.0059 (10)
C23	0.0176 (12)	0.0162 (12)	0.0139 (12)	−0.0009 (10)	0.0001 (10)	0.0002 (10)
C24	0.0165 (12)	0.0124 (11)	0.0172 (13)	−0.0013 (10)	0.0032 (10)	−0.0002 (10)
C25	0.0181 (13)	0.0154 (12)	0.0255 (14)	0.0025 (11)	0.0037 (11)	0.0076 (11)

### Geometric parameters (Å, º)

**Table:**

Br1—C4	1.903 (3)	C11—H11	0.9500
O1—C7	1.224 (3)	C12—C19	1.480 (3)
O2—C22	1.362 (3)	C13—C14	1.379 (4)
O2—C25	1.434 (3)	C13—C18	1.388 (4)
N1—C12	1.329 (3)	C14—C15	1.394 (3)
N1—N2	1.366 (3)	C14—H14	0.9500
N2—C11	1.347 (3)	C15—C16	1.384 (4)
N2—C13	1.435 (3)	C15—H15	0.9500
C1—C6	1.389 (4)	C16—C17	1.389 (4)
C1—C2	1.393 (4)	C16—H16	0.9500
C1—C7	1.504 (4)	C17—C18	1.392 (4)
C2—C3	1.383 (4)	C17—H17	0.9500
C2—H2	0.9500	C18—H18	0.9500
C3—C4	1.376 (4)	C19—C20	1.394 (3)
C3—H3	0.9500	C19—C24	1.391 (3)
C4—C5	1.382 (4)	C20—C21	1.385 (4)
C5—C6	1.394 (4)	C20—H20	0.9500
C5—H5	0.9500	C21—C22	1.391 (4)
C6—H6	0.9500	C21—H21	0.9500
C7—C8	1.470 (4)	C22—C23	1.400 (3)
C8—C9	1.328 (4)	C23—C24	1.383 (4)
C8—H8	0.9500	C23—H23	0.9500
C9—C10	1.450 (3)	C24—H24	0.9500
C9—H9	0.9500	C25—H25A	0.9800
C10—C11	1.382 (3)	C25—H25B	0.9800
C10—C12	1.427 (3)	C25—H25C	0.9800
			
C22—O2—C25	117.2 (2)	C14—C13—N2	119.2 (2)
C12—N1—N2	105.0 (2)	C18—C13—N2	119.4 (2)
C11—N2—N1	112.2 (2)	C13—C14—C15	119.4 (2)
C11—N2—C13	128.4 (2)	C13—C14—H14	120.3
N1—N2—C13	119.4 (2)	C15—C14—H14	120.3
C6—C1—C2	119.1 (2)	C16—C15—C14	120.2 (2)
C6—C1—C7	118.7 (2)	C16—C15—H15	119.9
C2—C1—C7	122.1 (2)	C14—C15—H15	119.9
C3—C2—C1	120.8 (3)	C15—C16—C17	119.7 (2)
C3—C2—H2	119.6	C15—C16—H16	120.1
C1—C2—H2	119.6	C17—C16—H16	120.1
C4—C3—C2	118.7 (3)	C16—C17—C18	120.7 (3)
C4—C3—H3	120.6	C16—C17—H17	119.7
C2—C3—H3	120.6	C18—C17—H17	119.7
C3—C4—C5	122.3 (2)	C13—C18—C17	118.6 (2)
C3—C4—Br1	118.9 (2)	C13—C18—H18	120.7
C5—C4—Br1	118.7 (2)	C17—C18—H18	120.7
C4—C5—C6	118.2 (2)	C20—C19—C24	118.3 (2)
C4—C5—H5	120.9	C20—C19—C12	119.7 (2)
C6—C5—H5	120.9	C24—C19—C12	121.8 (2)
C1—C6—C5	120.8 (3)	C21—C20—C19	121.8 (2)
C1—C6—H6	119.6	C21—C20—H20	119.1
C5—C6—H6	119.6	C19—C20—H20	119.1
O1—C7—C8	122.2 (2)	C20—C21—C22	119.3 (2)
O1—C7—C1	119.7 (2)	C20—C21—H21	120.3
C8—C7—C1	118.1 (2)	C22—C21—H21	120.3
C9—C8—C7	121.1 (2)	O2—C22—C21	124.8 (2)
C9—C8—H8	119.4	O2—C22—C23	115.6 (2)
C7—C8—H8	119.4	C21—C22—C23	119.6 (2)
C8—C9—C10	127.1 (2)	C24—C23—C22	120.2 (2)
C8—C9—H9	116.4	C24—C23—H23	119.9
C10—C9—H9	116.4	C22—C23—H23	119.9
C11—C10—C12	104.5 (2)	C23—C24—C19	120.8 (2)
C11—C10—C9	123.8 (2)	C23—C24—H24	119.6
C12—C10—C9	131.6 (2)	C19—C24—H24	119.6
N2—C11—C10	107.2 (2)	O2—C25—H25A	109.5
N2—C11—H11	126.4	O2—C25—H25B	109.5
C10—C11—H11	126.4	H25A—C25—H25B	109.5
N1—C12—C10	111.1 (2)	O2—C25—H25C	109.5
N1—C12—C19	118.7 (2)	H25A—C25—H25C	109.5
C10—C12—C19	130.2 (2)	H25B—C25—H25C	109.5
C14—C13—C18	121.4 (2)		
			
C12—N1—N2—C11	0.0 (3)	C9—C10—C12—C19	6.5 (5)
C12—N1—N2—C13	−179.2 (2)	C11—N2—C13—C14	−171.7 (2)
C6—C1—C2—C3	0.3 (4)	N1—N2—C13—C14	7.3 (3)
C7—C1—C2—C3	−177.2 (2)	C11—N2—C13—C18	8.5 (4)
C1—C2—C3—C4	0.2 (4)	N1—N2—C13—C18	−172.5 (2)
C2—C3—C4—C5	−0.8 (4)	C18—C13—C14—C15	1.4 (4)
C2—C3—C4—Br1	−178.9 (2)	N2—C13—C14—C15	−178.5 (2)
C3—C4—C5—C6	1.0 (4)	C13—C14—C15—C16	−1.1 (4)
Br1—C4—C5—C6	179.1 (2)	C14—C15—C16—C17	0.2 (4)
C2—C1—C6—C5	−0.1 (4)	C15—C16—C17—C18	0.6 (4)
C7—C1—C6—C5	177.5 (2)	C14—C13—C18—C17	−0.6 (4)
C4—C5—C6—C1	−0.5 (4)	N2—C13—C18—C17	179.2 (2)
C6—C1—C7—O1	−13.0 (4)	C16—C17—C18—C13	−0.3 (4)
C2—C1—C7—O1	164.4 (3)	N1—C12—C19—C20	−120.3 (3)
C6—C1—C7—C8	167.2 (2)	C10—C12—C19—C20	56.7 (4)
C2—C1—C7—C8	−15.4 (4)	N1—C12—C19—C24	54.7 (3)
O1—C7—C8—C9	−19.6 (4)	C10—C12—C19—C24	−128.3 (3)
C1—C7—C8—C9	160.2 (2)	C24—C19—C20—C21	0.1 (4)
C7—C8—C9—C10	179.5 (2)	C12—C19—C20—C21	175.2 (2)
C8—C9—C10—C11	−174.4 (3)	C19—C20—C21—C22	−0.6 (4)
C8—C9—C10—C12	1.9 (5)	C25—O2—C22—C21	4.0 (4)
N1—N2—C11—C10	0.3 (3)	C25—O2—C22—C23	−176.8 (2)
C13—N2—C11—C10	179.4 (2)	C20—C21—C22—O2	−179.9 (2)
C12—C10—C11—N2	−0.5 (3)	C20—C21—C22—C23	0.9 (4)
C9—C10—C11—N2	176.7 (2)	O2—C22—C23—C24	179.9 (2)
N2—N1—C12—C10	−0.3 (3)	C21—C22—C23—C24	−0.8 (4)
N2—N1—C12—C19	177.2 (2)	C22—C23—C24—C19	0.3 (4)
C11—C10—C12—N1	0.5 (3)	C20—C19—C24—C23	0.1 (4)
C9—C10—C12—N1	−176.3 (3)	C12—C19—C24—C23	−175.0 (2)
C11—C10—C12—C19	−176.6 (2)		

### Hydrogen-bond geometry (Å, º)

Cg1 is the centroid of the C1–C6 ring.

**Table:**

*D*—H···*A*	*D*—H	H···*A*	*D*···*A*	*D*—H···*A*
C11—H11···O1^i^	0.95	2.25	3.198 (3)	173
C25—H25*B*···*Cg*1^ii^	0.98	2.61	3.478 (3)	148

Symmetry codes: (i) −*x*+2, −*y*+1, −*z*+2; (ii) −*x*+2, −*y*+2, −*z*+1.
